# Effectiveness of rotavirus vaccines against hospitalisations in Japan

**DOI:** 10.1186/s12887-017-0916-7

**Published:** 2017-07-11

**Authors:** Yoshiyuki Fujii, Atsuko Noguchi, Shinobu Miura, Haruka Ishii, Toyoko Nakagomi, Osamu Nakagomi, Tsutomu Takahashi

**Affiliations:** 1 Department of Pediatrics, Akita University Graduate School of Medicine, Hondo 1-1-1, Akita, 010-8543 Japan; 2 Department of Pediatrics, Yuri-Kumiai General Hospital, 38 Kawaguti Aza Yago,Yurihonjo, Akita, 015-8511 Japan; 3 Center for Bioinformatics and Molecular Medicine, Nagasaki University, 1-12-4 Sakamoto, Nagasaki, 852-8523 Japan; 4 Department of Molecular Epidemiology, Graduate School of Biomedical Sciences, Nagasaki University, 1-12-4 Sakamoto, Nagasaki, 852-8523 Japan

**Keywords:** Rotavirus gastroenteritis, Rotavirus vaccine, Hospitalisation, Vaccine effectiveness, Case-control study, Screening method

## Abstract

**Background:**

In Japan, rotavirus hospitalisation occurs at a rate from 2.8 to 13.7 per 1000 child-years among children age less than 5 years, and it imposes a substantial burden to the healthcare system in the country. While both monovalent (RV1) and pentavalent (RV5) rotavirus vaccines are licensed in Japan, neither has been incorporated in the national infant immunization programme. In this study, we estimated vaccine effectiveness (VE) in Japan.

**Methods:**

This study was conducted in Yuri-Kumiai General Hospital located in a city in the north-western part of Japan. Age-eligible children for rotavirus vaccination were enrolled if they were hospitalized for rotavirus gastroenteritis between September 2013 and August 2016. Rotavirus gastroenteritis was defined by the detection of rotavirus antigen by immunochromatography. “Vaccinated” was defined as infant inoculated with at least one dose of either RV1 or RV5. A conditional logistic regression analysis was performed by modelling the year of birth, year of admission, residence of the children and vaccination status, and by matching the age of cases with that of test-negative controls. The adjusted odds ratio of the vaccinated over unvaccinated was then used to calculate VE in the formula of (1 – adjusted odds ratio) × 100.

**Results:**

Out of the 244 patients enrolled, rotavirus antigen was detected in 55 (22.5%) of whom 10 (18.2%) were vaccinated, whereas 94 (49.7%) of 189 test-negative controls were vaccinated. During the study period, the vaccine uptake rate in the controls increased from 36.2% to 61.8%. On the other hand, the vaccination coverage over the three years was 64.2% in Yuri-Honjo city (three quarters of the catchment), and 91.4% in Nikaho city (one quarter of the catchment). The VE was calculated to be 70.4% (95% confidence interval: 36.0–86.4%, *P* = 0.002). The point estimate of the VE was lower but its 95% confidence interval overlaps those of the efficacies obtained from clinical trials in Japan.

**Conclusion:**

The rotavirus vaccine was effective in the real-world setting in Japan as in the clinical trials, and the introduction of rotavirus vaccine in the national infant immunization schedule will substantially reduce the number of rotavirus gastroenteritis hospitalisation in Japan.

## Background

Rotavirus A causes severe acute gastroenteritis in infants and young children worldwide. In 2004, the last year before rotavirus vaccine was available, rotavirus was reported to cause an estimated 527,000 annual deaths worldwide [1]. Thus, in 2009 the World Health Organization (WHO) recommended that rotavirus vaccine should be incorporated in the infant immunization schedule of every country in the world (http://www.who.int/mediacentre/news/releases/2009/rotavirus_vaccines_20090605/en/). Although the number of deaths due to rotavirus gastroenteritis is small in Japan, the incidence rate of rotavirus hospitalisations was reported to range from 2.8 to 13.7 per 1000 child-years among children less than 5 years of age [2–7], imposing a substantial burden to the healthcare system in the country.

In the Yuri-Honjo district in Akita Prefecture, a locale in north-western Japan, where the present study was conducted, continuous epidemiological surveys have been carried out spanning over the last three decades [2–7]. These studies showed that the incidence of hospitalisation due to rotavirus gastroenteritis were 12.7–14.9/ 1000 child-years among children less than 5 years of age.

In bridging clinical trials conducted in Japan, RV1 showed efficacies of 79.3% (95% confidence interval [CI]: 60.5–89.8%) and 91.6% (95% CI: 62.4–99.1%) against any and severe rotavirus gastroenteritis, respectively [8]. Similarly, RV5 showed efficacies of 74.5% (95% CI: 39.9%–90.6%), 80.2% (95% CI: 47.4%–94.1%), and 100% (95% CI: 55.4%–100%) in preventing any severity, moderate-to-severe, and severe rotavirus-gastroenteritis [9]. Based on the results of these bridging clinical trials, RV1 and RV5 were licensed in Japan in November 2011 and July 2012, respectively, but neither vaccine has been incorporated in the infant immunization program.

In other high-income countries where rotavirus vaccines have already been incorporated in the national immunization schedule, significant reduction in the number of rotavirus hospitalisations have occurred [10], rendering rotavirus second to norovirus in aetiology of acute gastroenteritis among children in the United States of America [11]. In addition to the presence of large burden of rotavirus gastroenteritis in Japan and the availability of two rotavirus vaccines with proven efficacy in clinical trials, evidence that RV1 and RV5 work well in the real world setting will facilitate informed decision by policy makers to introduce rotavirus vaccine in the infant immunization schedule in Japan. Japan Paediatric Society recommends RV1 to be administered at 2 and 3 months of age, and RV 5 at 2, 3 and 4 months of age. In addition, the first dose of either vaccine is recommended to be administered between 6 weeks and 14 weeks and 6 days of age.

In Japan, the national childhood immunization schedule includes *Haemophilus influenzae* type B, pneumococcus, measles and rubella (but not mumps), Bacillus Calmette et Guerin, diphtheria-pertussis-tetanus-inactivated polio, Japanese encephalitis, varicella, and hepatitis B. No cost is borne by the parents for the vaccines included in the national immunization programme. By contrast, rotavirus vaccine remains in the private market and parents pay around 300 United States dollar equivalent for the full course. However, there are a few municipal governments that subsidise the immunization cost in part or in full.

Thus, the aim of this study was to estimate the combined effectiveness of the two rotavirus vaccines against rotavirus hospitalisation in a locale in northern part of Japan where there are much evidence of rotavirus disease burden. In this study, we evaluated vaccine effectiveness (VE) by a case-control design.

## Methods

This study was conducted in Yuri-Kumiai General Hospital located in the centre of a geographically-well defined administrative region of the south-western part of Akita prefecture, comprising Yuri-Honjo city (about three quarters of the hospital catchment) and Nikaho city (about one quarter of the hospital catchment). The hospital has 724 beds, of which 35 are allocated for paediatric patients, and they are the sole paediatric inpatient beds available in the region. Thus, it was assumed that virtually all children living in Yuri-Honjo and Nikaho cities with uncomplicated acute gastroenteritis would be admitted to this hospital.

This work was approved by the Institutional Review Board and Ethics Committee of Yuri-Kumiai General Hospital, and Akita University Graduate School of Medicine, Japan.

### Case control definition

Acute gastroenteritis is defined as three or more passages of looser-than-usual stool or watery diarrhoea during the preceding 24 h. Children were enrolled if they were hospitalised due to acute gastroenteritis between September 2013 and August 2016, lived in either Yuri-Honjo city or Nikaho city, were born after July 2011, were between 8 and 59 weeks of age at the time of hospitalisation, and consent was obtained from their parents or guardians.

Cases were defined as those who tested positive for rotavirus antigen and controls as those who tested negative. Rotavirus antigen test was done by the use of an immunochromatography kit (RapidTesta®Rota Adeno, Sekisui Medical Co. LTD., Tokyo, Japan), which has a sensitivity of 97% and a specificity of 100% (according to the manufacturer’s instructions).

“Vaccinated” was defined as infant inoculated with at least one dose of either RV1 or RV5, whose onset of acute gastroenteritis occurred after >14 days since vaccination (during which time protective immune responses were allowed to develop). Vaccination history was ascertained by vaccination record in the maternal handbook which had been provided (by law) to each pregnant mother in Japan.

The following children were excluded from the study. Children whose onset of acute gastroenteritis occurred after 48 h of hospital admission, and whose acute gastroenteritis persisting >14 days before admission were excluded in accordance with the generic protocol of the WHO (http://www.who.int/entity/immunization/monitoring_surveillance/burden/vpd/surveillance_type/sentinel/WHO_IVB_08.16_eng.pdf).

### Data analysis

The coverage of rotavirus vaccine in the community was calculated based on the vaccination record kept in the relevant department in the municipal government for those infants who were administered doses of rotavirus vaccine and the number of live birth in each calendar year (as a surrogate for the number of infants in each calendar year). Nikaho city has started full amount of financial assistance for rotavirus vaccine since April 2013, and Yuri-Honjo city also has subsidised one third of vaccination cost since April 2013. There was no policy change in subsidization in each of municipal governments throughout the study period.

The vaccination coverage was calculated by dividing the number of infants vaccinated with rotavirus vaccine that was obtained from the municipal administration offices of Yuri-Honjo city and Nikaho city by the number of live birth (as a surrogate for the number of infants in each year) in each city according to the vital statistics information.

The age at which subjects were hospitalised was categorised as follows; 0–2, 3–5, 6–11, 12–23, 24–59 months of age, and cases were matched with controls within these age categories. Thus, conditional multivariable logistic regression analysis was performed with Stata ver 13.1 (StataCorp, College Station, Texas, USA) to calculate the VE after controlling the years in which they were hospitalized and the cities where they lived.

## Results

**Fig. 1:**
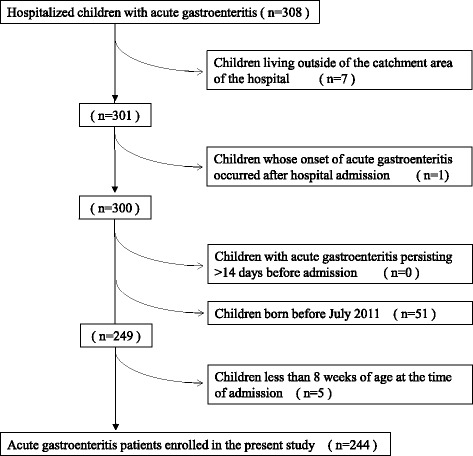
Flow diagramme from hospitalized children initially retrieved as having acute gastroenteritis to the final cases enrolled in the present study

Out of the 308 patients with acute gastroenteritis who were initially enrolled in the study, 244 met the inclusion criteria (Fig. 1). Rotavirus antigen was detected in 55 (22.5%) patients of whom 10 (18.2%) were vaccinated. On the other hand, 94 (49.7%) of 189 rotavirus-negative patients with acute gastroenteritis were vaccinated. However, the vaccine uptake rate in the rotavirus-negative patients increased during the study period: 36.2% in 2013/2014, 53.8% in 2014/2015, and 61.8% in 2015/2016 (Table 1).

**Table 1:** Number of vaccinated children and vaccine coverage in each season

Season		2013/9/1–2014/8/31	2014/9/1–2015/8/31	2015/9/1–2016/8/31	total
Number of vaccinated children among the number of rotavirus positive (coverage %)		5/32 (15.6%)	2/10 (20.0%)	3/13 (23.1%)	10/55(18.2%)
Number of vaccinated children among the number of rotavirus negative(coverage %)		25/69 (36.2%)	35/65 (53.8%)	34/55 (61.8%)	94/189(49.7%)
rotavirus positive gastroenteritis	vaccinated	5 (Y5, N0)	2 (Y2, N0)	3 (Y3, N0)	10
	not-vaccinated	27(Y24. N3)	8(Y6, N2)	10(Y10, N0)	45
	not-age-eligible	8	1	0	9
rotavirus negative gastroenteritis	vaccinated	25	35	34	94
	not-vaccinated	44	30	22	96
	not-age-eligible	34	11	2	47

When the vaccination coverage in each of the two cities was calculated based on the data obtained from each of the municipal governments, a substantial difference was noted (Table 2). On average, the coverage over the three year period in Yuri-Honjo city was 64.2%, whereas that in Nikaho city was 91.4% (Table 2).

**Table 2:** The vaccine uptake rates (coverage) in Yuri-Honjo city and Nikaho city, Akita Prefecture, Japan

a: Yuri-Honjo city			
	birth cohort	number of vaccinated infants	coverage (95% CI) %
2013	522	296	56.7 (52.3–61.0)
2014	477	300	62.9 (58.3–67.2)
2015	448	333	74.3 70.0–78.3)
total	1447	929	64.2 (61.7–66.7)
b: Nikaho city			
	birth cohort	number of vaccinated infants	coverage (95% CI) %
2013	131	120	91.6 (85.5–95.7)
2014	142	130	91.5 (85.7–95.6)
2015	146	133	91.1 (85.3–95.2)
total	419	383	91.4 (88.3–93.9)

**Fig. 2:**
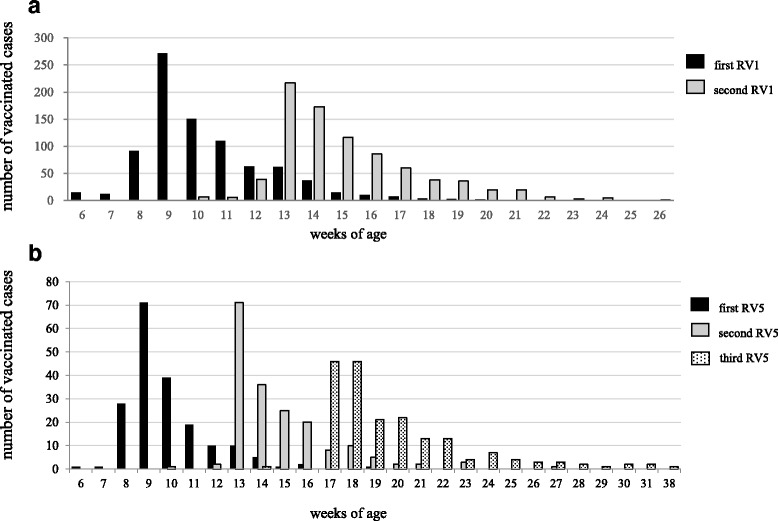
Age distribution at the time of rotavirus vaccinations. **a**: RV1. **b**: RV5. Black bar shows first vaccination of each type, grey bar shows second vaccination of each type, and dotted bar shows the third dose of RV5 vaccinination

The administrative data obtained from the municipal governments also allowed us to examine the adherence to the immunization schedule recommended by the Japan Paediatric Society (http://www.jpeds.or.jp/modules/en/index.php?content_id=7):i.e., 2 and 3 months of age for RV1, and 2, 3, and 4 months of age for RV5. For RV1 vaccination, 95% of the first dose was given during the period between 6 weeks and 14 weeks and 6 days, and 99% of the second dose was given during the period between 10 and 24 weeks of age (Fig. 2). For RV5 vaccination, 99% of each of the three doses was given during the period between 6 weeks and 14 weeks and 6 days, between 10 and 28 weeks of age, and between 14 and 32 weeks of age (Fig. 2). Thus, the high compliance was observed in the immunization practice in these two cities.

A conditional multivariable logistic regression analysis was performed as the rotavirus antigen positivity as binary outcome after matching the age categories of cases with those of test-negative controls and including vaccination status (at least one dose), residence (Yuri-Honjo city or Nikaho city), season (2013/2014, 2014/2015, or 2015/2016), and the year of birth as covariates. The analysis showed that the adjusted odds ratio of vaccinated over unvaccinated against hospitalisation due to rotavirus gastroenteritis was 0.296 with a 95% CI of 0.136–0.640 (*P* = 0.002). Thus, the combined effectiveness of rotavirus vaccines against hospitalisation due to rotavirus gastroenteritis among children less than 5 years of age was 70.4% with a 95% CI of 36.0–86.4%.

## Discussion

By using a case-control design, this study demonstrated that rotavirus vaccines prevented hospitalisation due to rotavirus gastroenteritis from children age-eligible for rotavirus vaccine in northern part of Japan where a high burden of rotavirus disease was well documented [4–7]. The point estimate of the VE value (70.4%) was slightly lower than the efficacies obtained in clinical trials for both RV1 and RV5, but given their 95% CI (36.0–86.4%), the effectiveness in the real world setting was consistent with the efficacies in Japan for both RV1 (92% [95%CI: 62–99] against severe rotavirus gastroenteritis) [8] and RV5 (81% [95%CI: 49.6–94.3] against moderate-to-severe rotavirus gastroenteritis) [9].

This study observed higher vaccine uptake rates in the community (64.2% and 91.4% for Yuri-Honjo and Nikaho, respectively) than in test-negative control patients (47.9% on average). The difference may partly result from the timing of vaccination (which depends on the age of the infants) and the timing of disease acquisition (which is substantially affected by the season). While the neighborhood controls are traditionally preferred, the test-negative controls are also well accepted in recent years in estimating rotavirus VE [12].

On the other hand, an observed difference in the vaccine uptake rates between the two cities was unlikely to be an artefact but probably reflected the different levels of financial support to the residents. To solve the disparity in vaccine uptake rates and to make rotavirus vaccine accessible by every infant in the country, it is important to expedite the incorporation of rotavirus vaccines in the national immunization schedule.

In Japan, there is a recent study conducted in Saga, a prefecture located in the Southern part of the country, in which the screening method was used to estimate the rotavirus VE [13]. They reported that the VE (both RV1 and RV5 combined) was 69.5% and 88.8% for clinically-diagnosed rotavirus gastroenteritis and rotavirus hospitalisation, respectively, although the VE was only statistically significant for rotavirus hospitalisation with a broad 95%CI (34.3–100%) [13]. Unlike Saga prefecture where no rotavirus disease burden study was done prior to vaccine use, it was shown that 13.7 (6.8–20.7)/ 1000 child-years among children less than 5 years of age living in Yuri-Honjo and Nikaho cities during the most recent 10 years before the introduction of rotavirus vaccines in the country [7]. Given the 70% VE, the burden of rotavirus hospitalisation will decrease to as small as 4.1 per 1000 child-years among children less than 5 years of age, if the rotavirus vaccines have fully been incorporated in the infant immunization schedule. Actually, according to the data obtained during the study period (data not shown), the hospitalisation rates due to acute rotavirus gastroenteritis in this area were 13.4/1000 child-years (2013/14), 3.1/1000 child-years (2014/15), and 5.1/1000 child-years (2015/16). Thus, substantial reduction of the rotavirus disease burden by fully implementing rotavirus vaccines is an achievable goal.

When the studies to estimate the rotavirus VE in high-income countries were reviewed, the VE values ranged from 68% to 98% with overlapping 95% CIs [14]; 96% (95% CI: 95–97%) in Austria [15], 90% (95%CI: 81–95%) in Belgium [16], 92%(95%CI: 50–98.7%) in Finland [17], 80%(95%CI: 77–83%) in Germany [18], 92%(95%CI: 60–99%) [19] and 80%(95%CI: 68–88%) in USA [20], 91%(95%CI: 62–98%) in Canada [21], 86%(95%CI: 78–91%) and 88%(95%CI: 81–92%) in Spain [22], and 96% (95%CI: 84–99%) in Portugal [23]. The 95% CI for the combined rotavirus VE obtained in this study overlaps the ranges shown above.

There are limitations to this study. First, the odds ratio was not adjusted for some potential risk factors such as attendance to nurseries and kindergarten, presence of siblings, other members in the household. Second, VE was estimated for at least one dose versus no dose, which might result in lower VE values. Third, the type of the vaccines administered was not distinguished but a combined VE value was calculated. Fourth, circulating genotypes during the study period were not fully examined. Fifth, the positive test results by immunochromatography were not validated by molecular assays; false positive results, if occurred, might affect VE estimates. Additionally, the possibility of picking up the vaccine strain by the immunochromatograhy kit might exist. Sixth, the number of case patients enrolled was less than required to obtain a tighter 95% CI; at least 94 cases were needed when the expected VE was 80% with a 95%CI of 60–90% and the vaccination coverage was 60% according to the WHO generic protocol.

## Conclusions

The rotavirus vaccine was effective in the real-world setting in Japan as in the clinical trial, and the introduction of rotavirus vaccine in the infant immunization schedule will likely reduce the number of rotavirus gastroenteritis hospitalisation substantially in Japan.

## Abbreviations

RV1: human monovalent vaccine containing G1P [8].
RV5: human bovine pentavalent vaccine containing G1-G4 and P [8].
VE: Vaccine effectiveness
